# Identification of a highly stable bioactive 3-hydroxyproline-containing tripeptide in human blood after collagen hydrolysate ingestion

**DOI:** 10.1038/s41538-022-00144-4

**Published:** 2022-06-03

**Authors:** Yuki Taga, Yu Iwasaki, Chisa Tometsuka, Noriko Funato, Yasutaka Shigemura, Masashi Kusubata, Kazunori Mizuno

**Affiliations:** 1 Nippi Research Institute of Biomatrix, 520-11 Kuwabara, Toride, Ibaraki 302-0017 Japan; 2 Department of Nutrition, Faculty of Domestic Science, Tokyo Kasei University, 1-18-1 Kaga, Itabashi-ku, Tokyo 173-8602 Japan; 3 Research Core, Tokyo Medical and Dental University, 1-5-45 Yushima, Bunkyo-ku, Tokyo 113-8510 Japan

**Keywords:** Peptides, Mass spectrometry

## Abstract

There are increasing reports demonstrating high bioavailability of 4-hydroxyproline (4Hyp)-containing oligopeptides after oral ingestion of collagen hydrolysate and their bioactivity. In contrast, no study investigates the fate of another collagen-specific but minor amino acid, 3Hyp. Here, we identified Gly-3Hyp-4Hyp tripeptide in human blood at high concentrations, comparable to other 4Hyp-containing oligopeptides, after ingesting porcine skin collagen hydrolysate. Additionally, Gly-3Hyp-4Hyp uniquely maintained the maximum concentration until 4 h after the ingestion due to its exceptionally high resistance to peptidase/protease demonstrated by incubation with mouse plasma. In mice, oral administration of collagen hydrolysate prepared from bovine tendon, which contains a higher amount of 3Hyp, further increased blood Gly-3Hyp-4Hyp levels compared to that from bovine skin. Furthermore, Gly-3Hyp-4Hyp showed chemotactic activity on skin fibroblasts and promoted osteoblast differentiation. These results highlight the specific nature of the Gly-3Hyp-4Hyp tripeptide and its potential for health promotion and disease treatment.

## Introduction

The oral bioavailability of bioactive peptides released from food proteins is important to exert their bioactivity in vivo^1^. Most of the orally ingested proteins/peptides are hydrolyzed into amino acids in the gastrointestinal tract and blood. However, recently, it has been revealed that various di- and tripeptides exist in the blood after oral ingestion of collagen hydrolysate^2–6^. The blood concentrations of collagen-derived oligopeptides reach µM levels, significantly higher than those of other food-derived peptides^7^. The most major collagen-derived peptide in the blood is prolyl-4-hydroxyproline (Pro-4Hyp)^2^, and the next is generally 4Hyp-Gly^3^. In addition, many types of oligopeptides, including Xaa-4Hyp (Xaa = any amino acids), Xaa-4Hyp-Gly, and Gly-Pro-4Hyp, have also been detected in the blood^4,6^. These peptides have various biological activities, such as stimulating the growth of skin fibroblasts^3,8^, promoting osteoblast differentiation^9,10^, and chemotactic activity on skin fibroblasts, tenocytes, and peripheral blood neutrophils^11–13^. The collagen-derived bioactive oligopeptides possessing high oral bioavailability are possibly the major contributing factor to the beneficial effects of collagen hydrolysate ingestion on bone^14^, joint^15^, skin^16^, and other targets^17^, which have been reported by many groups, particularly in recent years.

The common feature of those collagen-derived oligopeptides detected in the blood is the presence of 4Hyp within the sequence. Prolyl hydroxylation occurs uniquely in collagenous Gly-Xaa-Yaa repeat sequences (Xaa and Yaa are any amino acids). Pro residues at the Yaa position are mostly hydroxylated to 4Hyp, accounting for ~100 residues per 1000 amino acid residues in collagen^18^. Hydroxylation of Pro residues confers high resistance to peptidase/protease digestion of peptides^19^. Therefore, although orally ingested collagen hydrolysate is sequentially digested by gastrointestinal enzymes, some parts remain in the peptide form containing Hyp (~20–30% of total Hyp)^20^ and are transported into the blood mainly via peptide transporters in the intestinal brush-border membrane^21,22^. The absorbed Hyp-containing oligopeptides are distributed to various tissues and finally excreted into the urine^6,23,24^.

In addition to 4Hyp at the Yaa position, another form of Hyp, namely 3Hyp, is generated at the Xaa position of the Gly-Xaa-Yaa sequences only if 4Hyp is present at the adjacent Yaa position^25^. Prolyl 3-hydroxylation is a very minor modification in skin type I collagen (~1 residue/1000 residues); however, the modification rate largely varies with the source (tissue and species) and collagen type^25^. While absorption/metabolism and biological activity of 4Hyp-containing oligopeptides, especially Pro-4Hyp, have been extensively investigated, few studies have focused on 3Hyp-containing oligopeptides. The present study investigated the kinetics in blood, stability, and biological activity of Gly-3Hyp-4Hyp tripeptide identified to appear at high concentrations in human blood after oral ingestion of collagen hydrolysate.

## Results

### 3Hyp absorbed into the blood is mostly as the peptide form after oral ingestion of collagen hydrolysate

**Fig. 1:**
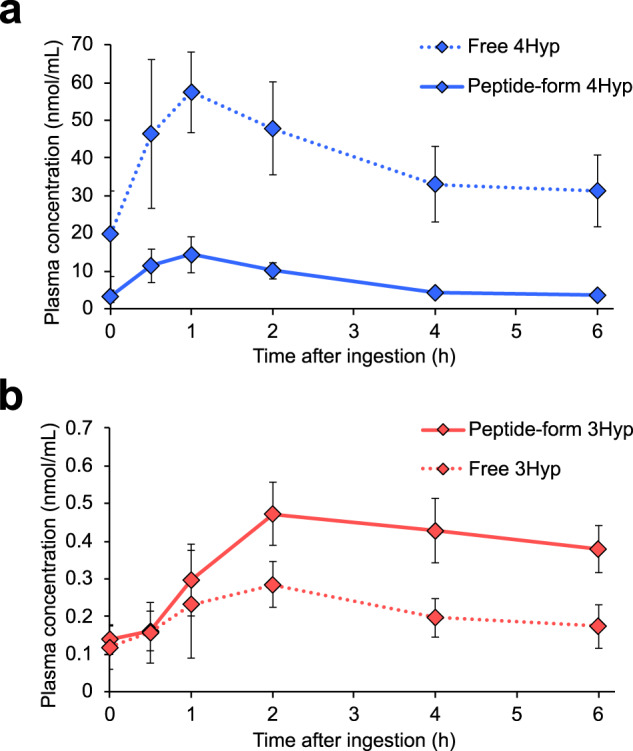
Concentrations of free and peptide-form Hyp in human plasma after oral ingestion of collagen hydrolysate. Human plasma samples collected before (0 h) and 0.5, 1, 2, 4, and 6 h after the ingestion of porcine skin collagen hydrolysate were analyzed by LC−MS in MRM mode with (total Hyp) or without (free Hyp) acid hydrolysis. Peptide-form Hyp was quantitated by subtracting the quantitative values of free Hyp from total Hyp. **a** 4Hyp and **b** 3Hyp were separately analyzed by APDS derivatization. The data represent the mean ± SD (*n* = 8).

We first analyzed concentration changes of free and peptide-form 4Hyp/3Hyp in human blood after oral ingestion of porcine skin collagen hydrolysate using LC–MS in multiple reaction monitoring (MRM) mode. We used a derivatization method using 3-aminopyridyl-*N*-hydroxysuccinimidyl carbamate (APDS)^26^ to separate 4Hyp and 3Hyp, otherwise difficult to discriminate in the biological fluid, as described previously^27^. After the ingestion of collagen hydrolysate, it was confirmed that free and peptide-form 4Hyp in plasma increased (Fig. 1a), and increases in both free and peptide-form 3Hyp were also detected (Fig. 1b). The majority of 4Hyp was in the free amino acid form, consistent with previous observations^2,4,20^. In contrast, the plasma levels of peptide-form 3Hyp were interestingly higher than those of free 3Hyp, although the concentrations were relatively low compared to those of peptide-form 4Hyp. Furthermore, the peak point of peptide-form 3Hyp was delayed compared to that of peptide-form 4Hyp (2 h vs. 1 h). Peptide-form 3Hyp largely maintained the maximum concentration until 6 h, while the blood concentration of peptide-form 4Hyp completely returned to the basal level after 4 h.

### Gly-3Hyp-4Hyp is the major peptide form of 3Hyp in the blood after oral ingestion of collagen hydrolysate

**Fig. 2:**
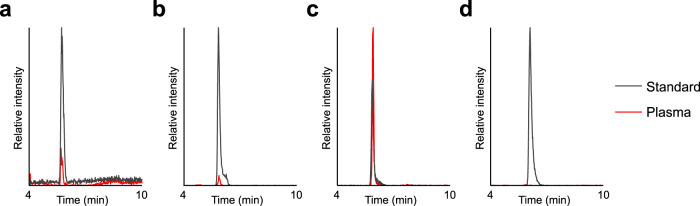
MRM chromatograms of 3Hyp-containing di- and tripeptides in human plasma after oral ingestion of collagen hydrolysate. **a** Gly-3Hyp, **b** 3Hyp-4Hyp, **c** Gly-3Hyp-4Hyp, and **d** 3Hyp-4Hyp-Gly were detected by LC–MS in MRM mode for respective standards (100 pg each) or deproteinized human plasma collected 1 h after the ingestion of porcine skin collagen hydrolysate.

We next sought to determine which kind of 3Hyp-containing oligopeptide is absorbed into the blood by using human plasma collected 1 h after the ingestion of porcine skin collagen hydrolysate. Since 3Hyp is known to be present at the Xaa position of the Gly-Xaa-4Hyp sequence^25^, we selected Gly-3Hyp, 3Hyp-4Hyp, Gly-3Hyp-4Hyp, and 3Hyp-4Hyp-Gly as conceivable di- and tripeptides containing 3Hyp. Using synthetic standards of those peptides as references, we found a high peak of Gly-3Hyp-4Hyp in the plasma sample on MRM analysis (Fig. 2c), while in others, only a slight or no peak was observed (Fig. 2a, b, and d). This finding suggested that most of the peptide-form 3Hyp appeared in blood after ingesting collagen hydrolysate is Gly-3Hyp-4Hyp. We confirmed that the peak observed in the plasma was certainly Gly-3Hyp-4Hyp, not Gly-4Hyp-4Hyp, by chromatographic discrimination of these peptides after APDS derivatization (Supplementary Fig. 1).

### Gly-3Hyp-4Hyp shows persistently high blood levels after oral ingestion of collagen hydrolysate

**Fig. 3:**
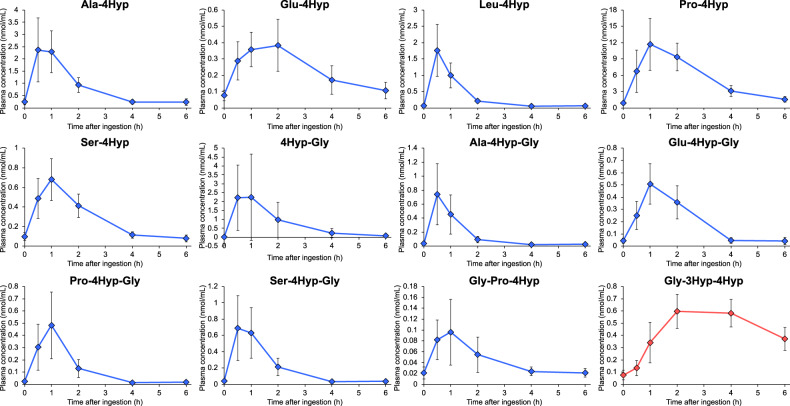
Concentrations of Hyp-containing oligopeptides in human plasma after oral ingestion of collagen hydrolysate. Human plasma samples collected before (0 h) and 0.5, 1, 2, 4, and 6 h after the ingestion of porcine skin collagen hydrolysate were analyzed by LC−MS in MRM mode. The data represent the mean ± SD (*n* = 8).

To evaluate the kinetic profile in the blood, we analyzed Gly-3Hyp-4Hyp and other major 4Hyp-containing oligopeptides, which were selected based on the previous data^6^, in the time-course human plasma samples by MRM analysis. A previously developed internal standard mixture of collagen-derived oligopeptides (SI-digest)^4^ was used for accurate quantitation. After ingesting porcine skin collagen hydrolysate, all the analyzed peptides were increased in plasma, and the most major oligopeptide in the blood was Pro-4Hyp (Fig. 3), as reported previously^2,4,6^. Gly-3Hyp-4Hyp showed an obviously different profile to other 4Hyp-containing oligopeptides. The time to maximum concentration (*T*_max_) of Gly-3Hyp-4Hyp was delayed compared to that of other oligopeptides (2.63 h vs. 0.63–1.44 h; Table 1), consistent with the observation for peptide-form 3Hyp (Fig. 1). While the concentrations of most of the di- and tripeptides returned to the initial level at 4–6 h after the ingestion, that of Gly-3Hyp-4Hyp remained high at the time points. The maximum concentration (*C*_max_) of Gly-3Hyp-4Hyp reached the submicromolar level, which was comparable to other 4Hyp-containing oligopeptides, except for several peptides such as Pro-4Hyp. Due to the persistently high plasma levels, the area under the concentration−time curve (AUC_0–6 h_) of Gly-3Hyp-4Hyp resulted in the fourth highest value, next to that of Pro-4Hyp, Ala-4Hyp, and 4Hyp-Gly. The concentrations and profile of Gly-3Hyp-4Hyp matched with those estimated as the total of peptide-form 3Hyp in Fig. 1b, which indicates that most of the 3Hyp-containing oligopeptides absorbed into the blood were this tripeptide form.

**Table 1:** AUC_0-6 h_, *C*_max_, and *T*_max_ of Hyp-containing oligopeptides in human plasma after oral ingestion of collagen hydrolysate.

	AUC_0-6 h_ (nmol/mL h)	*C*_max_ (nmol/mL)	*T*_max_ (h)
Ala-4Hyp	5.10 ± 1.63	2.60 ± 1.14	0.88 ± 0.23
Glu-4Hyp	1.46 ± 0.39	0.47 ± 0.14	1.44 ± 0.62
Leu-4Hyp	2.12 ± 0.57	1.83 ± 0.72	0.63 ± 0.23
Pro-4Hyp	34.18 ± 9.92	12.16 ± 4.33	1.38 ± 0.52
Ser-4Hyp	1.71 ± 0.35	0.69 ± 0.21	1.06 ± 0.42
4Hyp-Gly	4.82 ± 4.76	2.52 ± 2.36	0.75 ± 0.27
Ala-4Hyp-Gly	0.93 ± 0.46	0.77 ± 0.42	0.63 ± 0.23
Glu-4Hyp-Gly	1.19 ± 0.23	0.54 ± 0.13	1.25 ± 0.46
Pro-4Hyp-Gly	0.76 ± 0.38	0.49 ± 0.27	0.94 ± 0.18
Ser-4Hyp-Gly	1.25 ± 0.49	0.78 ± 0.37	0.75 ± 0.27
Gly-Pro-4Hyp	0.27 ± 0.13	0.10 ± 0.06	0.81 ± 0.26
Gly-3Hyp-4Hyp	2.77 ± 0.58	0.62 ± 0.14	2.63 ± 1.19

The data represent the mean ± SD (*n* = 8).

### Gly-3Hyp-4Hyp has extremely high stability

**Fig. 4:**
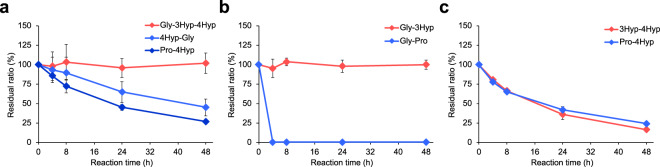
Stability of 3Hyp-containing peptides in mouse plasma. Synthetic oligopeptide mixtures, including **a** Gly-3Hyp-4Hyp, 4Hyp-Gly, and Pro-4Hyp, **b** Gly-3Hyp and Gly-Pro, or **c** 3Hyp-4Hyp and Pro-4Hyp, were incubated with mouse plasma at 37 °C for 4, 8, 24, and 48 h. The residual ratio at each time point was calculated using the peak area ratio relative to the control (0 h) on LC−MS analysis in MRM mode. The data represent the mean ± SD (*n* = 3).

We hypothesized that the unique kinetic profile of Gly-3Hyp-4Hyp in the blood was due to its high stability because the presence of Hyp within the sequence confers peptidase/protease resistance^19^. Synthetic peptides were incubated with mouse plasma at 37 °C to assess the stability as performed in previous studies^19,28^. 4Hyp-Gly and Pro-4Hyp, both reported to have high stability^3^, were gradually reduced and finally to less than half of the initial amount after 48 h of incubation (Fig. 4a). In contrast, Gly-3Hyp-4Hyp was completely intact during the reaction period, demonstrating its extremely high resistance to peptidase/protease digestion. We further analyzed two sets of peptides (Gly-3Hyp and Gly-Pro; 3Hyp-4Hyp and Pro-4Hyp) to clarify which sequence is essential for the stability of Gly-3Hyp-4Hyp. While Gly-Pro was rapidly diminished in the mouse plasma, Gly-3Hyp remained unchanged during the reaction (Fig. 4b). In contrast, 3Hyp-4Hyp was gradually degraded as well as Pro-4Hyp (Fig. 4c). These results indicate that 3Hyp located at the middle position mainly contributes to the extremely high stability of Gly-3Hyp-4Hyp.

### Oral administration of tendon collagen hydrolysate results in higher absorption of Gly-3Hyp-4Hyp

Type I collagen present in tendon has a higher amount of 3Hyp, which increases during the tissue development stage^29^, relative to that from skin and bone^30^. Therefore, it was expected that ingestion of tendon collagen hydrolysate results in further higher blood levels of Gly-3Hyp-4Hyp. To verify this, we purified collagens from bovine skin and tendon to perform oral administration experiments using mice. The purified skin and tendon collagens similarly had high amounts of 4Hyp (~110 residues/1000 residues; Supplementary Table 1), while the 3Hyp content in the tendon collagen was low (3.2 residues/1000 residues) but fourfold higher than that in the skin collagen (0.8 residues/1000 residues), consistent with the previous results^30^.

**Fig. 5:**
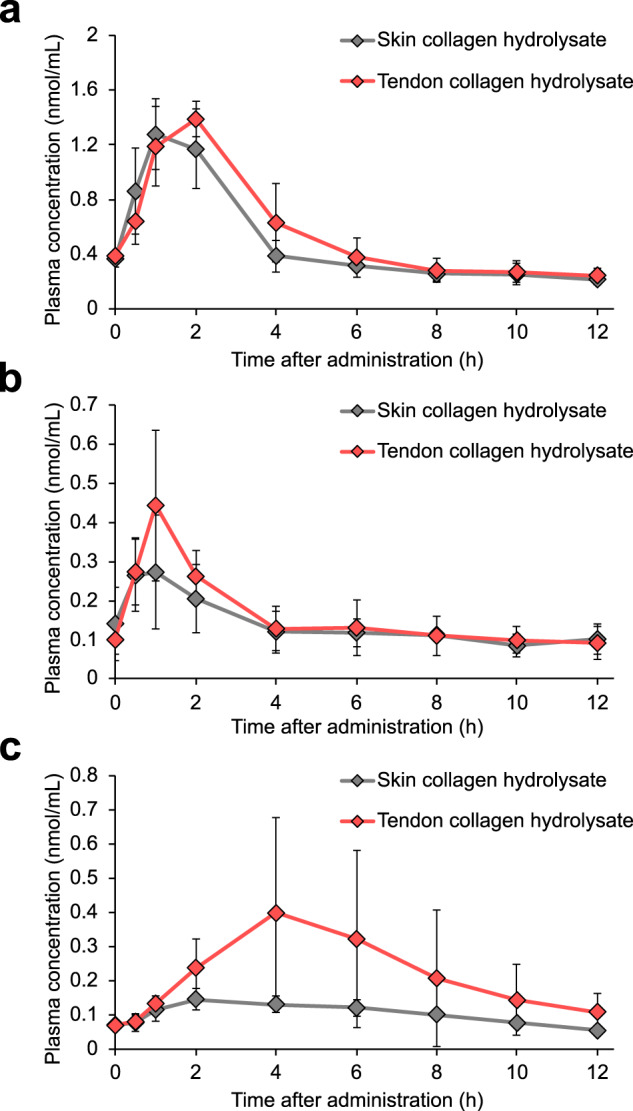
Concentrations of Hyp-containing oligopeptides in mouse plasma after oral administration of collagen hydrolysate prepared from bovine skin or tendon. Mouse plasma samples collected before (0 h) and 0.5, 1, 2, 4, 6, 8, 10, and 12 h after the administration of bovine skin or tendon collagen hydrolysate were analyzed by LC−MS in MRM mode for quantitation of **a** Pro-4Hyp, **b** 4Hyp-Gly, and **c** Gly-3Hyp-4Hyp. The data represent the mean ± SD (*n* = 5).

The skin and tendon collagens were further hydrolyzed with pancreatin, and the average molecular weight of the prepared collagen hydrolysates was confirmed to be similar (1880 and 1771 Da, respectively). We monitored the peptide levels in plasma until 12 h after administering the two collagen hydrolysates to totally evaluate the kinetic profile of the long-lasting peptide. In the case of Pro-4Hyp and 4Hyp-Gly, there were no significant differences between the administration of the skin and tendon collagen hydrolysates (Fig. 5a, b). In contrast, plasma concentrations of Gly-3Hyp-4Hyp were apparently increased in mice administered the tendon collagen hydrolysate (Fig. 5c). At 12 h after the administration, Gly-3Hyp-4Hyp still did not appear to reach the baseline level. Significant differences between the two collagen hydrolysates were detected for the AUC_0-12 h_ and *C*_max_ of Gly-3Hyp-4Hyp, and the AUC_0–12 h_ became higher than that of 4Hyp-Gly when the tendon collagen hydrolysate was administered (Table 2). These results demonstrate that the plasma level of Gly-3Hyp-4Hyp can be increased significantly depending on the source of the administered collagen hydrolysate.

**Table 2:** AUC_0–12 h_, *C*_max_, and *T*_max_ of Pro-4Hyp, 4Hyp-Gly, and Gly-3Hyp-4Hyp in mouse plasma after oral administration of collagen hydrolysate prepared from bovine skin or tendon.

	Skin collagen hydrolysate			Tendon collagen hydrolysate		
	AUC_0-12 h_ (nmol/mL h)	*C*_max_ (nmol/mL)	*T*_max_ (h)	AUC_0–12 h_ (nmol/mL h)	*C*_max_ (nmol/mL)	*T*_max_ (h)
Pro-4Hyp	5.88 ± 0.55	1.38 ± 0.23	1.40 ± 0.55	6.79 ± 1.08	1.44 ± 0.18	1.60 ± 0.55
4Hyp-Gly	1.65 ± 0.29	0.31 ± 0.12	1.00 ± 0.61	1.92 ± 0.50	0.45 ± 0.19	0.90 ± 0.22
Gly-3Hyp-4Hyp	1.26 ± 0.16	0.15 ± 0.03	2.80 ± 1.10	2.73 ± 1.72*	0.41 ± 0.27*	3.60 ± 0.89

^*^P < 0.05 compared to skin collagen hydrolysate (Student’s *t* test). The data represent the mean ± SD (*n* = 5).

### Gly-3Hyp-4Hyp has chemotactic activity on skin fibroblasts and promotes osteoblast differentiation

**Fig. 6:**
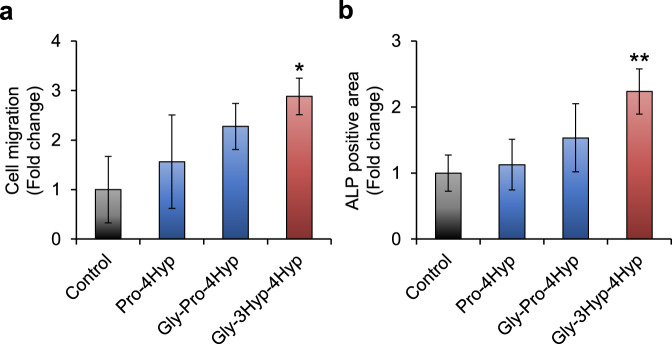
Fibroblast chemotaxis and osteoblast differentiation induced by Gly-3Hyp-4Hyp. **a** Migration of human skin fibroblasts through the gelatin-coated filter membrane of the transwell insert was evaluated after 18 h of incubation with 0 (control) or 200 nmol/mL of synthetic peptides (Pro-4Hyp, Gly-Pro-4Hyp, or Gly-3Hyp-4Hyp) added to the lower chamber (*n* = 3). The migrated cells were labeled with calcein-AM and counted by fluorescence microscopy. **b** Osteoblast differentiation was evaluated by ALP staining after culturing mouse pre-osteoblast MC3T3-E1 cells with 0 (control) or 200 nmol/mL of synthetic peptides (Pro-4Hyp, Gly-Pro-4Hyp, or Gly-3Hyp-4Hyp) for 6 days (*n* = 6). *P < 0.05 and **P < 0.01 compared to the control (ANOVA/Dunnett’s test). The data represent the mean ± SD.

Since the most major collagen-derived oligopeptide, Pro-4Hyp, is reported to promote cell migration of skin fibroblasts and differentiation of osteoblasts^9,11^, we investigated whether Gly-3Hyp-4Hyp has such biological activities at a concentration of 200 nmol/mL, which was determined based on the previous observation that the maximum blood concentration of collagen-derived peptides reached up to this concentration in some human subjects after ingesting collagen hydrolysate (0.385 g/kg of body weight)^8,20^. In the culture of human skin fibroblasts using the transwell system, Gly-3Hyp-4Hyp added to the bottom chamber significantly enhanced cell migration through the filter membrane compared to the control (~2.9-fold; Fig. 6a and Supplementary Fig. 2). Although Pro-4Hyp and Gly-Pro-4Hyp, both previously exhibited fibroblast chemotactic activity at relatively high concentrations (2.5 and 12.5 µmol/mL)^11^, seemed to increase migrated cells, those alterations were not statistically significant under our experimental conditions. The effect of Gly-3Hyp-4Hyp on differentiation of mouse pre-osteoblasts MC3T3-E1 was evaluated by alkaline phosphatase (ALP) activity and cell mineralization. Similar to the chemotaxis assay, the ALP activity was significantly increased by Gly-3Hyp-4Hyp (~2.2-fold) compared to the control but not by other peptides (Fig. 6b). Although an increase in ALP activity of MC3T3-E1 cells with Pro-4Hyp was previously observed by our and other groups^9,10^, statistical significance was not detected for the peptide in this study. Additionally, only Gly-3Hyp-4Hyp significantly enhanced mineralization (Supplementary Fig. 3).

## Discussion

A growing number of studies report the effectiveness of collagen hydrolysate ingestion on improving disease states, including osteoporosis^14^, osteoarthritis^15^, pressure ulcer^31^, hypertension^32^, and depression^33^, and also on promoting skin health^16^. Although the mechanism of which had been unclear, in 2005, 4Hyp-containing oligopeptides were found to appear in blood at markedly high concentrations after ingesting collagen hydrolysate^2^. After that, links between the beneficial effects of collagen hydrolysate ingestion and bioactivities of 4Hyp-containing oligopeptides have been increasingly established mainly through in vitro experiments^3,8–13,19,34,35^. Furthermore, several animal studies have provided direct evidence that 4Hyp-containing oligopeptides, especially Pro-4Hyp, exhibit their bioactivities in vivo after oral administration^34–37^. In the present study, we found that 3Hyp-containing tripeptide, Gly-3Hyp-4Hyp, shows persistent high blood concentrations after oral ingestion of collagen hydrolysate. It was demonstrated that Gly-3Hyp-4Hyp has high chemotactic activity on skin fibroblasts and promotional activity of osteoblast differentiation. Given the high blood concentration and the potent bioactivities, Gly-3Hyp-4Hyp possibly functions as a significant contributor to the beneficial effects of collagen hydrolysate ingestion.

We showed that Gly-3Hyp-4Hyp possesses extremely high resistance to peptidase/protease by incubating with mouse plasma, while Pro-4Hyp and 4Hyp-Gly were slowly degraded. We speculate that aminopeptidase P is a critical enzyme for the slow but gradual degradation of collagen-derived oligopeptides, except for Gly-3Hyp-4Hyp. Aminopeptidase P is ubiquitously found in tissues and body fluids, and catalyzes the cleavage of N-terminal X-Pro bonds, which are difficult to cleave by other aminopeptidases^38^. Although aminopeptidase P almost specifically recognizes penultimate Pro, it was reported that small amounts of Pro and Hyp were generated from Gly-Pro-Hyp^39^, indicating that this enzyme potentially degrades Pro-4Hyp. In addition, slight cleavage of the Gly-Hyp bond of Gly-Hyp-Ala was also observed in the study (either 4Hyp or 3Hyp was not mentioned). These observations suggest that aminopeptidase P can attack N-terminal X-Hyp bonds, albeit to a lesser extent. The plasma degradation assay demonstrated that the high stability of Gly-3Hyp-4Hyp is mainly due to the Gly-3Hyp sequence. However, in contrast to the complete stability of Gly-3Hyp dipeptide, 3Hyp-4Hyp dipeptide was gradually degraded in the plasma as well as Pro-4Hyp, suggesting that 3Hyp at the carboxy side of the cutting site can confer even higher resistance to enzymatic digestion than 4Hyp. Despite the potential degradation of the 3Hyp-4Hyp sequence, Gly-3Hyp-4Hyp remains intact in the presence of the stable N-terminal dipeptide sequence, Gly-3Hyp.

Sontakke et al. reported that Gly-Pro-4Hyp was efficiently transported across the intestinal Caco-2 cell monolayer rather than Pro-4Hyp, suggesting the involvement of active transporters in the intestinal permeation of this type of collagenous tripeptide^22^. Gly-3Hyp-4Hyp may be efficiently absorbed into the blood across the wall of the intestine in a similar way. Yazaki et al. indicated that collagen-derived oligopeptides, such as Pro-4Hyp and Gly-Pro-4Hyp, were incorporated into the mouse skin after oral administration of collagen hydrolysate^5^. In addition, several studies reported cellular uptake of collagen-derived oligopeptides, such as by skin fibroblasts^40^, osteoblasts^10^, and tenocytes^12^. Tenocytes are suggested to internalize Pro-4Hyp through multiple pathways, including integrin-mediated endocytosis^12^. Collagen-derived oligopeptides delivered to tissues and subsequently internalized into cells would then exert their bioactivities by interacting with specific partners, such as transcription factors^41^. The accumulation of Gly-3Hyp-4Hyp in tissues after the ingestion of collagen hydrolysate and the internalization efficiency of this tripeptide into cells require investigations in future works.

The abundance of 3Hyp was approximately 1/140 of 4Hyp in skin type I collagen (Supplementary Table 1). However, after porcine skin collagen hydrolysate was ingested, Gly-3Hyp-4Hyp appeared in human blood at high concentrations, which were comparable to other 4Hyp-containing di- and tripeptides. Consistent with previous observations^2,4,20^, the major portion of 4Hyp detected in the blood after the ingestion was the free amino acid form. In contrast, we revealed that almost all of the 3Hyp absorbed into the blood was in the form of Gly-3Hyp-4Hyp due to its exceptionally high stability. This is the reason for the high concentrations of Gly-3Hyp-4Hyp in the blood despite the low abundance of 3Hyp in the ingested collagen. Our data indicate the remarkable oral bioavailability of this unique peptide.

The 4Hyp content hardly differs depending on the collagen source since this major form of Hyp is essential for maintaining the thermal stability of collagen^42,43^. In contrast, the 3Hyp content dramatically varies with the tissue, species, and collagen type, although its biological role has remained unclear^25^. We demonstrated that oral administration of pancreatin hydrolysate of tendon collagen, in which the 3Hyp content is higher than that in skin collagen, significantly increased the AUC and *C*_max_ of Gly-3Hyp-4Hyp. This result suggests that selecting other sources containing further larger amounts of 3Hyp leads to more high absorption of Gly-3Hyp-4Hyp comparable or surpassing that of Pro-4Hyp. We recently analyzed collagens from various species and showed that the 3Hyp content is particularly high in invertebrate collagens^43^, which can be candidates for the source to prepare Gly-3Hyp-4Hyp-rich collagen hydrolysate.

It should be noted that endogenous tissue collagen could also generate non-negligible amounts of collagen-derived oligopeptides, especially where tissue reconstruction occurs^44^. It was reported that contents of Pro-4Hyp, Leu-4Hyp, and Gly-Pro-4Hyp in the mouse ear were significantly increased by 2,4-dinitrofluorobenzene-induced dermatitis compared to vehicle-treated another ear^44,45^. In addition, an increase in Pro-4Hyp was also observed after wounding in mouse skin^46^. The local increases in collagen-derived oligopeptides in the inflammatory and wound healing sites suggest that those peptides generated during tissue remodeling exert their biological activities associated with the events. Indeed, a recent study reported that daily intraperitoneal injection of Pro-4Hyp decreased scar tissue formation in the skin of wound healing model mice^47^. It is reasonable that collagen-producing cells, such as skin fibroblasts and osteoblasts, are regulated by collagen-derived oligopeptides in an autocrine-like manner in response to tissue damage. In addition, in the case of Gly-3Hyp-4Hyp, type IV collagen may also be the major supplier of the peptide in a paracrine- or endocrine-like manner to those cells, since the basement membrane collagen contains higher amounts of 3Hyp (more than 10 residues/1000 residues)^48^. 3Hyp is a rare post-translationally modified amino acid in collagen, and its biological significance is little understood^25^. However, in view of the stability and bioactivity of Gly-3Hyp-4Hyp, 3Hyp possibly functions as a signal generated after degradation of tissue collagen in the form of the tripeptide.

It has been long known that collagen-derived oligopeptides, including synthetic peptides and bacterial collagenase digests, are chemoattractive for various cells in vitro^11–13,49,50^, which also suggests substantial involvement of degradation products of collagen in wound healing. Our data showed that Gly-3Hyp-4Hyp has chemotactic activity on skin fibroblasts. Hyp is suggested to be an essential constituent for collagen-derived peptides to function as chemotactic stimuli for skin fibroblasts^11^. Therefore, the presence of two consecutive Hyp may be important for the chemotactic activity of Gly-3Hyp-4Hyp. Indeed, the corresponding tripeptide with Pro at the middle position, Gly-Pro-4Hyp, did not show a statistically significant chemotactic response. The difference in the stability during incubation with cells may also affect the outcome. Similar discussions can be applied to the promotional activity of osteoblast differentiation by Gly-3Hyp-4Hyp observed here. Our in vitro data suggest the therapeutic potential of this bioactive tripeptide for damage in skin and bone, both showing beneficial effects of collagen hydrolysate ingestion in several clinical trials^14,16,31^. Although Pro-4Hyp deposited in tissue after oral administration of the peptide is reported to be further converted to amino acids and other metabolites^24,45^, Gly-3Hyp-4Hyp would more effectively function in tissue owing to the exceptionally high stability. Further studies are needed to clarify the fate and usage efficiency of the bioactive 3Hyp-containing tripeptide derived from orally ingested or endogenous collagen.

## Methods

### Materials and reagents

*trans*-4-Hydroxy-L-proline, *trans*-3-hydroxy-L-proline, pepsin, β-glycerophosphate disodium salt hydrate, L-ascorbic acid, and nitroblue tetrazolium chloride (NBT)/5-bromo-4-chloro-3-indolyl phosphate p-toluidine salt (BCIP) working solution were purchased from Sigma-Aldrich (St. Louis, MO, USA), APDS, pancreatin, and α-Modified minimal essential medium (α-MEM) were purchased from Wako Chemicals (Osaka, Japan), FluoroBlok 24-well plate insert (8.0 µm pore size) and 24-well companion plate were purchased from Corning (Corning, NY, USA), phenol red-free DMEM, GlutaMAX supplement, and dialyzed fetal bovine serum (FBS) were purchased from Thermo Fisher Scientific (Waltham, MA, USA), calcein-AM was purchased from Dojindo (Kumamoto, Japan), and gelatin from pig skin (MediGelatin) was procured from Nippi (Tokyo, Japan). Gly-Pro, Pro-4Hyp, 4Hyp-Gly, and Gly-Pro-4Hyp were purchased from Bachem (Bubendorf, Switzerland), and other peptides, including Gly-3Hyp-4Hyp, were custom synthesized by AnyGen (Gwangju, Korea).

### Ethics statement

All animal and human studies were approved by the Experimental Ethical Committee of Nippi Research Institute of Biomatrix.

### Preparation of time-course human plasma samples after oral ingestion of collagen hydrolysate

The human study was performed under the supervision of medical doctors following the Helsinki Declaration. Eight healthy volunteers who provided written informed consent fasted for 12 h before the experiment and then ingested 5 g of porcine skin collagen hydrolysate (PS-1, an average molecular weight of 3000−5000 Da; Nippi) dissolved in 100 mL of water. Venous blood was collected from the cubital vein before (0 h) and 0.5, 1, 2, 4, and 6 h after the ingestion. Plasma was prepared by centrifugation of the blood at 10,000 *g* for 10 min at 4 °C and stored at −80 °C until used for analysis.

### Quantitation of free and peptide-form Hyp in plasma

Free and peptide-form 4Hyp/3Hyp were analyzed by subtraction of quantitative values before and after acid hydrolysis as described previously^4^. In brief, the human plasma samples were deproteinized by adding three volumes of ethanol after mixing with acid hydrolysate of stable isotope-labeled collagen (SI-collagen) as an internal standard^4^. A portion of the ethanol-soluble fraction was dried using a centrifugal evaporator CVE-3100 (EYELA, Tokyo, Japan) to analyze free Hyp. Another portion was subjected to acid hydrolysis (6 N HCl/1% phenol, 110 °C for 20 h in the gas phase under N_2_) to analyze total Hyp (sum of free Hyp and peptide-form Hyp). The two types of samples were dissolved in 0.1 M sodium borate buffer (pH 8.8) and derivatized with APDS at 60 °C for 10 min as described previously^27^. The derivatized samples were analyzed by LC–MS in MRM mode using a 3200 QTRAP hybrid triple quadrupole/linear ion trap mass spectrometer (AB Sciex, Foster City, CA, USA) coupled to an Agilent 1200 Series HPLC system (Agilent Technologies, Palo Alto, CA, USA). The chromatographic separation was performed using a Hypercarb column (3 µm particle size, L × I.D. 100 mm × 2.1 mm; Thermo Fisher Scientific) at a flow rate of 400 µL/min and a column temperature of 80 °C with a binary gradient as follows: 100% solvent A (0.4% formic acid) for 2.5 min, linear gradient of 0–50% solvent B (100% acetonitrile) for 7.5 min, 90% solvent B for 2.5 min, and 100% solvent A for 2.5 min. The MRM transitions of APDS-4Hyp/3Hyp and APDS-^13^C_5_^15^N_1_-4Hyp/^13^C_5_^15^N_1_-3Hyp are shown in Supplementary Table 2. The plasma concentration of 4Hyp/3Hyp was determined using the peak area ratio of the nonlabeled analytes relative to the corresponding stable isotopically labeled analytes whose concentrations in the acid hydrolysate of SI-collagen were predetermined. The concentration of peptide-form Hyp was estimated by subtracting the concentration of free Hyp from total Hyp^4^.

### Analysis of Hyp-containing oligopeptides in plasma

The ethanol-soluble fraction of the deproteinized human plasma samples was dried using the centrifugal evaporator. For quantitative analysis of collagen-derived oligopeptides in the time-course samples, SI-digest prepared by sequential protease digestion of SI-collagen was mixed into the samples as an internal standard before the drying procedure^4^. The samples were reconstituted with 0.1% formic acid and analyzed by LC–MS in MRM mode using an Ascentis Express F5 HPLC column (5 µm particle size, L × I.D. 250 mm × 4.6 mm; Supelco, Bellefonte, PA, USA)^6^. The MRM transitions of Hyp-containing oligopeptides are shown in Supplementary Table 2. The plasma concentration of those oligopeptides was calculated by the peak area ratio of nonlabeled analytes relative to the corresponding stable isotopically labeled analytes whose concentrations in SI-digest were predetermined. AUC was calculated using the trapezoidal rule.

### Peptidase/protease resistance assay using mouse plasma

The stability of collagen-derived oligopeptides was assessed as described previously^19,28^. In brief, synthetic peptide mixtures (Gly-3Hyp-4Hyp/4Hyp-Gly/Pro-4Hyp, Gly-3Hyp/Gly-Pro, and 3Hyp-4Hyp/Pro-4Hyp) were mixed with fresh plasma prepared from male ICR mice at 6 months of age (Japan SLC, Shizuoka, Japan) at a concentration of 20 µg/mL each. The samples were incubated at 37 °C for 4, 8, 24, and 48 h and then deproteinized by ethanol precipitation. The ethanol-soluble fraction was diluted with 0.1% formic acid and analyzed by LC–MS in MRM mode using the Ascentis Express F5 HPLC column as described above.

### Oral administration experiments of pancreatin-hydrolyzed bovine skin and tendon collagen hydrolysates using mice

Collagen was purified from the skin of an 18-month-old bovine (raised in Hokkaido, Japan) or Achilles tendon of a 30-month-old bovine (Tokyo Shibaura Zoki, Tokyo, Japan). The tissues were digested with pepsin (5 mg/mL in 0.5 M acetic acid) at 4 °C overnight after defatting in ethanol. The extracted collagens were purified by salt precipitation (0.7 M NaCl), and the precipitates were dissolved in 5 mM acetic acid. The content of Pro, 4Hyp, and 3Hyp expressed as residues/1000 amino acid residues was quantitated by LC–MS in MRM mode after acid hydrolysis with SI-collagen used as an internal standard as reported previously^30^.

The skin and tendon collagens were denatured to gelatin at 60 °C for 1 h and digested with pancreatin (1:100 enzyme/substrate ratio) at 37 °C and pH 8.1 (adjusted by NaOH) for 16 h. After heating at 100 °C for 5 min to deactivate the enzyme, the average molecular weight of the prepared collagen hydrolysates was determined by size exclusion chromatography using a Superdex 30 Increase 10/300 GL column (GE Healthcare, Piscataway, NJ, USA) as described previously^28^. The pancreatin-hydrolyzed collagen hydrolysates were used for oral administration experiments using male ICR mice at 7 months of age (Japan SLC). A day before the procedure, their normal diet was replaced with a collagen-free diet (AIN-93M; Oriental Yeast, Tokyo, Japan). The mice were administered the skin collagen hydrolysate or tendon collagen hydrolysate (500 μL of 40 mg/mL solution dissolved in water, *n* = 5 each) using gastric sonde. Blood was collected from the tail vein before (0 h) and 0.5, 1, 2, 4, 6, 8, 10, and 12 h after the administration. The plasma concentration of Pro-4Hyp, 4Hyp-Gly, and Gly-3Hyp-4Hyp was measured by LC–MS in MRM mode after ethanol deproteinization with SI-digest as described above.

### Fibroblast chemotaxis assay

The migration assays were conducted using the FluoroBlok transwell system. Phenol red-free DMEM was supplemented with 2% GlutaMAX. Before the experiment, adult human skin fibroblasts (Kurabo, Osaka, Japan) were incubated with phenol red-free DMEM without FBS overnight. The FluoroBlok 24-well plate insert was placed in the 24-well companion plate, and 75 µL of 100 µg/mL gelatin solution was added and dried in the insert at room temperature overnight to coat the filter membrane. The bottom chamber was then filled with phenol red-free DMEM containing 200 nmol/mL of Pro-4Hyp, Gly-Pro-4Hyp, or Gly-3Hyp-4Hyp without FBS. As a control, distilled water was added in equal volume to the peptide solutions. FBS added at a concentration of 2% was used as a positive control, and significant cell migration was confirmed (data not shown). Cells suspended in phenol red-free DMEM without FBS were seeded onto the filter membrane of the insert (4 × 10^4^ cells/well). After incubation for 18 h, the cells that migrated and adhered to the lower surface of the filter were labeled with 5 µM calcein-AM added to the bottom chamber for 30 min in the incubator. The labeled cells were detected by a BZ-X800 fluorescence microscope (Keyence, Osaka, Japan) at 470/525 nm (Ex/Em). Seven measurement areas at 100 × magnification were arbitrarily selected, and the number of migrated cells whose size was >200 µm^2^ was counted using the Hybrid Cell Count application (Keyence analysis software).

### Osteoblast differentiation assay

The effect of collagen-derived oligopeptides on osteoblast differentiation was assessed by ALP activity and mineralization of the mouse pre-osteoblast cell line MC3T3-E1 (Riken Cell Bank, Ibaraki, Japan) as described previously^10^. In brief, for ALP assay, MC3T3-E1 cells were cultured in osteogenic differentiation medium (α-MEM containing 10% dialyzed FBS, 10 mM β-glycerophosphate, and 100 µg/mL L-ascorbic acid) with 200 nmol/mL of Pro-4Hyp, Gly-Pro-4Hyp, or Gly-3Hyp-4Hyp for 6 days. After washing, fixing, and permeabilizing the cells, NBT/BCIP working solution was added to stain the cells. Positive ALP staining areas were measured using ImageJ software (NIH; Bethesda, MD, USA). For the mineralization assay, MC3T3-E1 cells were similarly cultured in the osteogenic differentiation medium with 200 nmol/mL of Pro-4Hyp, Gly-Pro-4Hyp, or Gly-3Hyp-4Hyp for 7 days. Mineralization was measured by alizarin red staining using a calcification evaluation set (Cosmo Bio, Tokyo, Japan) according to the manufacturer’s instructions. The absorbance at 450 nm of the dye solution extracted from the cells was measured with a FLUOstar OPTIMA-6 microplate reader (BMG Labtech, Ortenberg, Germany).

### Statistical analysis

Statistical significances were evaluated by the one-sided Student’s *t* test using Microsoft Excel 2010 (Microsoft Corporation, Redmond, WA, USA) in the mouse oral administration experiments or by one-way ANOVA followed by Dunnett’s multiple-comparison test using GraphPad Prism version 4.0c for Macintosh (GraphPad Software, San Diego, CA, USA) in the fibroblast chemotaxis and osteoblast differentiation experiments. *P* values less than 0.05 were considered statistically significant. All data are expressed as the mean ± SD.

## Supplementary information

Supplementary Information

## Data Availability

The data that support the findings of this study are available from the corresponding author upon reasonable request.
