# Supplementary material for: Identification of a highly stable bioactive 3-hydroxyproline-containing tripeptide in human blood after collagen hydrolysate ingestion
Source: NPJ Sci Food. 2022 Jun 3;6:29. doi: 10.1038/s41538-022-00144-4 (PMC9166765; doi:10.1038/s41538-022-00144-4)
Supplement: Supplementary file 1 — Supplementary Information [file 41538_2022_144_MOESM1_ESM.pdf]

SUPPLEMENTARY INFORMATION

Identification of a highly stable bioactive 3-hydroxyproline-containing tripeptide in human blood after collagen hydrolysate ingestion

Yuki Taga[1, *], Yu Iwasaki[2], Chisa Tometsuka[1], Yasutaka Shigemura[2], Noriko Funato[3], Masashi Kusubata[1], and Kazunori Mizuno[1]

[1]Nippi Research Institute of Biomatrix, 520-11 Kuwabara, Toride, Ibaraki 302-0017, Japan

[2]Department of Nutrition, Faculty of Domestic Science, Tokyo Kasei University, 1-18-1 Kaga, Itabashi-ku, Tokyo 173-8602, Japan

[3]Research Core, Tokyo Medical and Dental University, 1-5-45 Yushima, Bunkyo-ku, Tokyo 113-8510, Japan

*Corresponding Author

Nippi Research Institute of Biomatrix, 520-11 Kuwabara, Toride, Ibaraki 302-0017, Japan

Tel: +81-297-71-3046; Fax: +81-297-71-3041

E-mail: y-taga@nippi-inc.co.jp

Table of Contents

Supplementary Figure 1: Separation of Gly-3Hyp-4Hyp and Gly-4Hyp-4Hyp by APDS derivatization.

Supplementary Figure 2: Representative fluorescence images of migrated human fibroblasts in a chemotaxis assay.

Supplementary Figure 3: Mineralization of osteoblasts cultured with Gly-3Hyp-4Hyp.

Supplementary Table 1: Pro and Hyp content in collagens purified from bovine skin and tendon (residues/1000 residues).

Supplementary Table 2: MRM transitions of analytes and their internal standards.

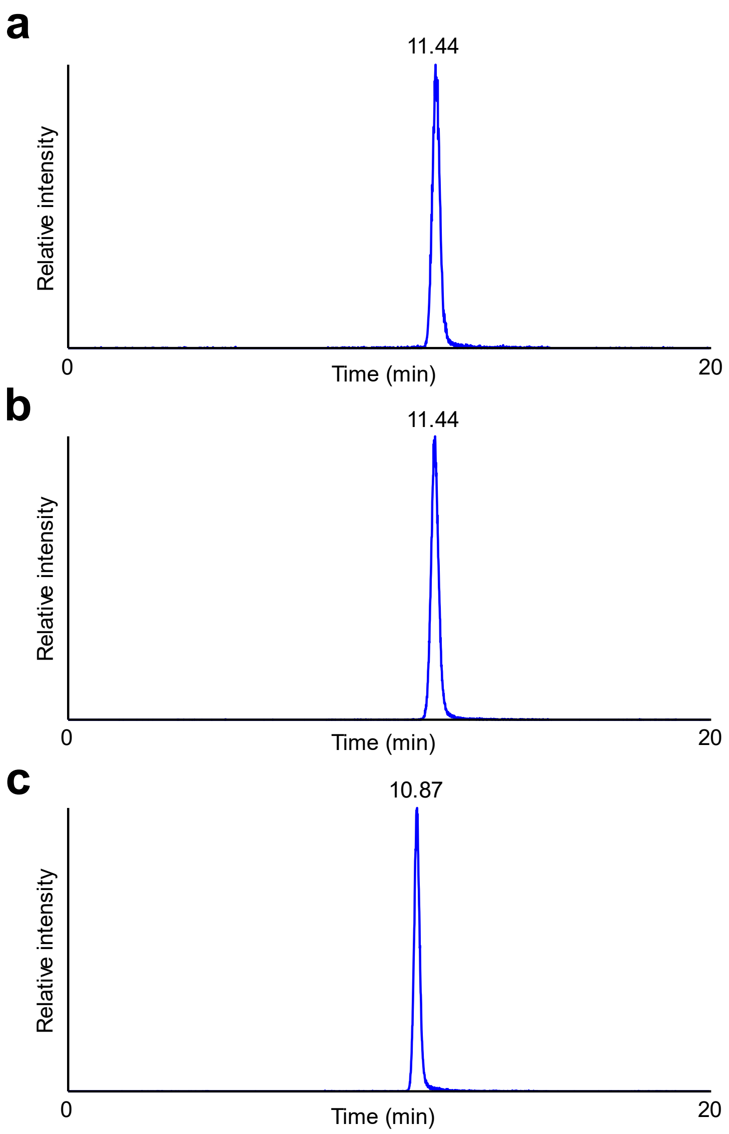

Supplementary Figure 1. **Separation of Gly-3Hyp-4Hyp and Gly-4Hyp-4Hyp by APDS derivatization.** (a) Ethanol-deproteinized human plasma collected 1 h after ingesting porcine skin collagen hydrolysate and standards of (b) Gly-3Hyp-4Hyp and (c) Gly-4Hyp-4Hyp were derivatized with APDS. APDS-derivatized Gly-3Hyp-4Hyp/Gly-4Hyp-4Hyp (*m/z* 422.1→143.2) were detected by MRM analysis with chromatographic separation using the Hypercarb column. Based on the retention time, the peak observed in the plasma sample can be judged as Gly-3Hyp-4Hyp.

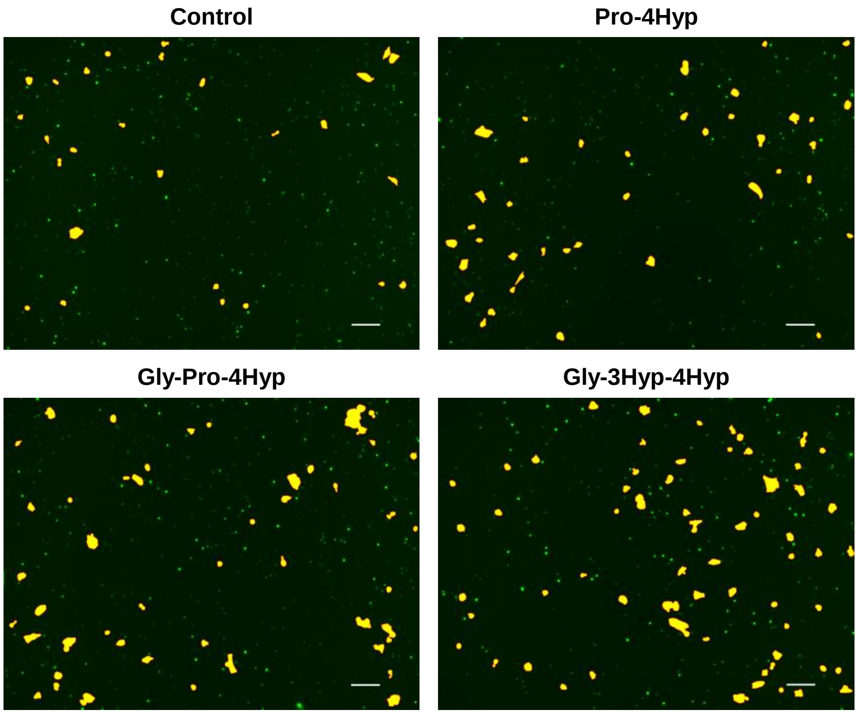

Supplementary Figure 2. Representative fluorescence images of migrated human fibroblasts in a chemotaxis assay. The lower limit of the count size was set at 200 μm^2 to exclude the filter's pores, through which fluorescence of cells on the upper surface of the filter was detected. Counted cells are shown by yellow. The scale bars represent 100 μm.

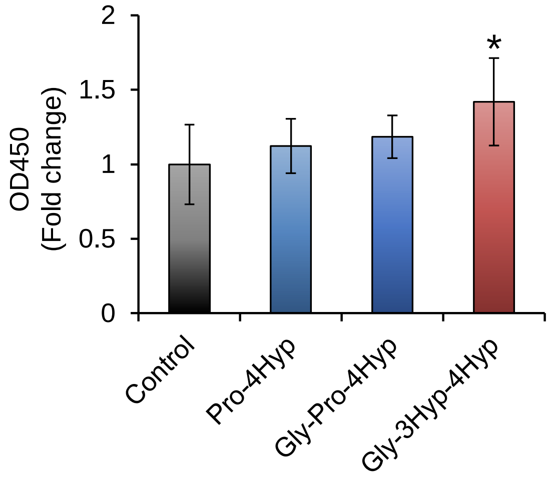

Supplementary Figure 3. **Mineralization of osteoblasts cultured with Gly-3Hyp-4Hyp.** Calcium deposits in the mineralized matrix were analyzed by alizarin red staining after culturing MC3T3-E1 cells with 0 (control) or 200 nmol/mL of Pro-Hyp, Gly-Pro-4Hyp, or Gly-3Hyp-4Hyp for 7 days. *$P < 0.05$ compared to the control (ANOVA/Dunnett's test). The data represent the mean ± SD (n = 6).

Supplementary Table 1. Pro and Hyp content in collagens purified from bovine skin and tendon (residues/1000 residues)

	Skin collagen	Tendon collagen
Pro	139.6 (55.1%)	132.1 (53.4%)
4Hyp	113.0 (44.6%)	112.1 (45.3%)
3Hyp	0.8 (0.3%)	3.2 (1.3%)

The data represent the mean of triplicate measurements. Values in parentheses represent the relative levels of Pro, 4Hyp, and 3Hyp.

Supplementary Table 2. MRM transitions of analytes and their internal standards

	Q1	Q3	Collision energy
APDS-4Hyp/3Hyp	252.2	121.1	33
SI-APDS-4Hyp/3Hyp	258.2	121.1	33
Ala-4Hyp	203.1	132.2	17
SI-Ala-4Hyp	209.1	138.2	17
Glu-4Hyp	261.2	243.2	15
SI-Glu-4Hyp	267.2	249.2	15
Leu-4Hyp	245.1	132.2	17
SI-Leu-4Hyp	251.1	138.2	17
Pro-4Hyp	229.2	70.1	37
SI-Pro-4Hyp	241.2	75.1	37
Ser-4Hyp	219.1	132.2	19
SI-Ser-4Hyp	225.1	138.2	19
4Hyp-Gly	189.1	86.1	21
SI-4Hyp-Gly	195.1	91.1	21
Ala-4Hyp-Gly	260.1	189.1	19
SI-Ala-4Hyp-Gly	266.1	195.1	19
Glu-4Hyp-Gly	318.2	86.1	41
SI-Glu-4Hyp-Gly	324.2	91.1	41
Pro-4Hyp-Gly	286.2	189.1	21
SI-Pro-4Hyp-Gly	298.2	195.1	21
Ser-4Hyp-Gly	276.2	189.1	19
SI-Ser-4Hyp-Gly	282.1	195.1	19
Gly-Pro-4Hyp	286.0	127.2	21
SI-Gly-Pro-4Hyp	298.0	132.2	21
Gly-3Hyp-4Hyp	302.2	143.2	23
SI-Gly-3Hyp-4Hyp	314.2	148.2	23
Gly-Pro	173.1	116.1	17
Gly-3Hyp	189.2	132.2	17
3Hyp-4Hyp	245.2	86.1	31
3Hyp-4Hyp-Gly	302.1	189.2	23
